# Gender differences in general and specialty outpatient mental health service use for depression

**DOI:** 10.1186/1471-244X-14-135

**Published:** 2014-05-09

**Authors:** Sarah Gagné, Helen-Maria Vasiliadis, Michel Préville

**Affiliations:** 1 Faculty of Medicine and Health Sciences, Université de Sherbrooke, Sherbooke, Canada; 2 Charles Lemoyne Research Centre, 150, place Charles-LeMoyne, Bureau 200, Longueuil, (Québec) J4K 0A8, Canada

**Keywords:** Gender, Mental health service use, Depression

## Abstract

**Background:**

This study ascertain gender-specific determinants of outpatient mental health (MH) service use for depression to highlight gender disparities in barriers to care and explain how depressed men and women in need of care might differ in their help-seeking behaviour.

**Methods:**

Data used in this study came from the Canadian Community Health Survey on Mental Health and Well Being, cycle 1.2 (CCHS 1.2) conducted by Statistics Canada in 2002 (*N =* 36,984). The sample was limited to respondents filling the criteria for a probable major depression in the 12 months prior to the interview (n = 1743). Gender-specific multivariate logistic regression analyses were carried out.

**Results:**

The results showed that 54.3% of respondents meeting criteria for major depression had consulted for mental health reasons in the year prior to interview. When looking at type of outpatient mental health service use, males were more likely to consult a general practitioner and a mental health specialist in the past year as opposed to females. However, females were more likely to consult a general practitioner only as opposed to no service use than males. Gender specific differences in determinants associated with outpatient service use included for males, lower adjusted household income, and for females, a younger age, the presence of social support, self-reported availability barriers, the presence of self-reported suicidal thoughts or attempt and a poorer self- perceived mental health.

**Conclusions:**

It is concluded that continued efforts to promote access to mental health care are needed for men and women affected by depression, and this, to target specific vulnerable populations and increase utilization rates.

## Background

Major depression is a significant public health issue and is a major cause of disability worldwide [1]. Community based surveys estimate 12-month prevalence rates ranging from 2.9% to 3.6% among men and 5.0% to 6.9% among women in the general population [2,3]. Not only is this highly prevalent mental disorder associated with increased mental health service use and increased costs due to treatment and reduced productivity due to days lost from work [4-9] it is also associated with decreased quality of life and social functioning, and increased mortality, primarily from suicide [7,10,11]. Although many treatments are available and have shown their efficacy for major depression, roughly half of depressed individuals will not seek professional help [12-23], and up to two-thirds will remain untreated or receive inappropriate or inadequate treatment [24-30]. Failure to receive treatment for those depressed individuals in need of care represents a major public health concern.

Many population-based studies have focused on reporting the determinants of service use for mental health reasons. One recurrent factor associated with mental health service use in the general population is female gender [20,22,31-37]. Studies focusing on populations with depression have shown inconsistent findings with regards to gender. Some have reported gender differences in the likelihood of seeking mental health care [14,18,38,39] whereas others have not [15,16,19,20,23,40-43]. An earlier study, focusing on gender differences by type of mental health service use, showed that females were more likely to consult general medical services, but not specialised mental health services [31]. Using data from a Canadian population based community sample of adults aged 18–65 years old, Drapeau and colleagues (2009) showed that women were more likely to use general medical services, psychological services and psychiatric services and this after controlling for the presence of major depression [44]. The authors, focusing on social roles, showed that women in employed work were more likely to use the services of psychologists whereas men who were in employed work were less likely to use general and psychiatric services. In another study using the same Canadian population based sample, Vasiliadis and colleagues (2009) showed that the odds of consulting with a family physician only, a family physician and a psychologist, and a family physician and other mental health professional (nurse, social worker, counsellor) but not a psychiatrist in the past year were larger in females than males [45]. Other studies have shown greater use of mental health specialised services in men [22,35].

Although most studies have shown gender differences in mental health service use, the majority of these studies were not designed to highlight gender disparities in barriers to care and to examine how depressed men and women might differ in their help-seeking behaviour. An earlier study carried out by Albizu-Garcia and colleagues (2001) in a community sample of adults inhabiting poor areas in Puerto Rico reported that males were more likely to use services when exhibiting morbidity and a poorer self-reported mental health, whereas women were more likely to consult mental health services if they had a history of prior mental health treatment [46]. In the general population, others have also shown that social and cultural factors and those related to acceptability issues (i.e. stigma against mental illness, health care providers, the health care system) and the perception and recognition of symptoms, have a differential impact on help seeking behaviours among men and women [31,44,47-49]. However, these studies also suggest that these factors may have less of an impact on the decision to use services when the disorder reaches a clinical threshold [31,44,47-49]. Using data from a community based survey focusing on Latinos and African Americans with major depression, Ojeda and colleagues (2006) reported gender specific determinants of outpatient mental health service use and found that older age was a barrier to service use in depressed males, and low education attainment in depressed females [18]. The results also showed that ethnic/racial minorities were barriers to service use in both males and females. Moreover, the authors also studied the effect of social and individual factors such as perceived need or stigma, financial barriers, health system barriers, general access barriers and social barriers to care. Gender differences however were not detected here. Given the specific populations of these studies the results cannot be generalised to other populations.

Given the high individual, societal and economic burden associated with depression, and given that many depressed men and women don’t seek help, it is imperative that better approaches are found to encourage men and women with needs to seek care. The gender specific analyses of the determinants of service use among depressed men and women can highlight disparities in service use and barriers to care which can help inform public mental health campaigns aimed at stigma against mental illness, and the negative perception of mental health providers and the system. Further, in Canada, mental health action plans have called for common mental disorders to be treated in primary care with the support of mental health specialists [50]. Studies have also shown better perceived care and perceived mental health needs being met when general practitioners (GP)/family physicians (FP) and mental health practitioners are consulted [51]. The objectives of this study were to therefore carry out gender-specific analyses and to ascertain the determinants of outpatient mental health service use among individuals with probable depression in a representative sample of Canadian community living residents. Using Andersen’s behavioural model of health service use, we studied predisposing, enabling/impeding, and need factors that have been identified in the literature. We also studied past year outpatient health service use for depression by type of providers consulted: (i) GP/FP only; (ii) GP/FP and other mental health specialist (psychiatrist, psychologist, social worker, nurse, and counsellor/psychotherapist); (iii) mental health specialist only.

## Methods

### Population

This study used data from the Canadian Community Health Survey on Mental Health and Well Being, cycle 1.2 (CCHS 1.2) conducted by Statistics Canada in 2002 (*N =* 36,984). The CCHS 1.2 was a cross-sectional survey of a nationally representative sample of non-institutionalized individuals aged 15 years and older living in private dwellings. The survey was designed to assess the prevalence and determinants of mental disorders, such as major depression, in the population and to document mental health service use, including antidepressant use, in Canada. Respondents were randomly selected using a stratified, multistage, clustered area sample. The overall response rate for the CCHS 1.2 in Canada was 77%. A detailed description of the method of selection of household interviews is reported elsewhere design [52].

The study protocol conformed to the Helsinki declaration of ethics and consent. The study protocol was reviewed and approved by the ethics committee of the University of Sherbrooke. All participants provided a signed informed consent.

### Study sample

In this study, the sample was limited to respondents filling the criteria for a probable major depression (n = 1743) in the 12 months prior to interview, which represented a prevalence of major depression reaching 4.8% overall, and 5.9% for females and 3.7% for males. In the CCHS 1.2, the presence of psychiatric disorders was measured using the computer-assisted version of the modified WMH-CIDI interview [51]. Questions were based on a Canadian adaptation of the World Health Organisation’s CIDI as used in the World Mental Health (WMH) project [53]. The WMH-CIDI assessed mental health profiles based on the definitions and criteria as outlined in the Diagnostic and Statistical Manual of Mental Disorders, Fourth Edition (DSM-IV) [54].

For the presence of a probable major depressive episode (MDE): Participants were first screened by questions, which asked about mood (sad, empty, or depressed), being discouraged or having a loss of interest in most things usually enjoyed, at any time in the person’s lifetime. Those answering positive to any item were then included in the depression module to assess whether they met criteria for depression. Participants who were screened into the depression module were then asked a series of symptom-related questions. Respondents who experienced a lifetime major depressive episode reported the following CCHS 1.2/WMH-CIDI criteria: 1) a period of two weeks or more with depressed mood or loss of interest or pleasure; 2) at least five additional symptoms (significant weight loss/gain or change in appetite, insomnia or hypersomnia, psychomotor agitation or retardation, fatigue or loss of energy, feelings of worthlessness, diminished ability to think or concentrate, recurrent thoughts of death); 3) clinically significant distress or social or occupational impairment; and 4) the symptoms are not better accounted for by bereavement. Respondents who experienced a major depressive episode in the past 12 months and reported a 12-month episode marked with impairment in occupational or social functioning were included in our study sample.

### Measures

#### Outcome variable

The outcome variable was past year mental health service use which was ascertained by questions that focused on service use for mental health reasons about emotions, mental health or use of alcohol or drugs. Past year service use (yes/no) in this paper was defined as using at least one outpatient resource for problems concerning emotions, mental health or use of alcohol or drugs in the 12 months prior to the interview. Specifically, outpatient service use was determined using self-reported questions assessing whether the respondent had ever consulted for mental health reasons a professional such as a GP/FP, psychologist, psychiatrists, other physician specialist, nurse, social worker, and counsellors. We did not consider in this study mental health service use during a hospitalisation or emergency department visit.

#### Respondent’s mental health status

For the purpose of this analysis, respondents with a past year major depressive episode were categorized as those having a ‘somatic depression’ versus those with a ‘pure depression’ [55]. Somatic depression was defined as having experienced all of the following symptoms for at least 2 weeks nearly every day: 1) appetite disturbance (loss/weight gain, change in appetite), 2) fatigue or loss of energy, 3) sleep disturbance (trouble falling asleep, staying asleep or waking too early or sleeping more than usual). A significant weight change was defined as 10 pounds or 4 kg. Weight gain due to pregnancy and weight loss due to illness were excluded. Respondents with ‘pure depression’ met criteria for MDE, but did not report all 3 somatic symptoms mentioned.

Among the need factors, we also considered the presence of self-reported suicide thoughts or attempts during the past year. Other mental disorders ascertained in the CCHS 1.2 such as the presence (yes/no) of anxiety disorders (AD), which included the presence of agoraphobia, social phobia, or panic disorder, and substance dependence of alcohol or illicit drugs in the past 12 months were also included. Self-perceived mental health was included in the analyses as excellent/very good versus good/fair/poor.

#### Respondent’s physical health status

The presence (Yes/No) of a chronic condition was also considered based on a record of 36 chronic medical conditions (i.e. cardiovascular, metabolic, respiratory diseases, etc.) qualified as “long-term conditions that are expected to last or have already lasted 6 months or more that have been diagnosed by a health professional”.

#### Enabling factors

Barriers to mental health service use were measured using 3 questions relating to whether the respondent reported a perceived unmet mental health care need due to accessibility (yes/no), acceptability (yes/no), or availability (yes/no) factors. Accessibility factors included cost, lack of transportation or issues such as childcare or scheduling. Availability barriers included waiting too long, help not available in area or at the time required. Acceptability issues included competing demands on their time, individuals chose to do without mental health care because of their attitude towards the illness, health care providers or the health care system. Examples include: deciding not to bother, not getting around to it, preferred to manage it themselves, didn’t think it could help, afraid to ask or language problems.

Social support was also studied and included tangible, emotional and informational. The presence of at least one of these sources of social support for most or all of the time in the past year was considered.

Adjusted household income for the number of people living in the household was retained and included two categories (high versus low).

#### Predisposing factors

The socio-demographic variables included in the analyses were: age (15–24, 25–49, 50–64, and 65 years and greater); education (less than high school versus high school and higher level attained); and marital status (married/common law versus separated/widowed/divorced/single).

### Analyses

In order to assess the determinants of service use, we used gender specific logistic regression to model past year outpatient mental health service use (yes/no) as a function of the predisposing, enabling and need study variables. We also carried out gender specific multinomial logistic regression analyses to determine, among outpatient service users, the factors associated with past year use of (i) GP/FP only versus GP/FP and other mental health specialists, and use of (ii) GP/FP only versus other mental health specialists only. With regards to missing data, there were no significant gender differences. All data presented were weighted according to Statistics Canada to deal with the complex sampling strategy [56]. The data were analyzed using SPSS version 18.0, and the total variance explained by the model was calculated using Nagelkerke Pseudo-R square.

## Results

The characteristics of the sample by gender are presented in Table 1. The multinomial regression analyses showed that the odds of consulting a GP/FP and a mental health specialist in the past year as opposed to a GP/FP only was 1.4 times larger in males than females (1.41 OR; 95% CI: 1.02 – 1.94). Further, the odds of consulting a GP/FP only as opposed to not consulting was 1.4 times larger in females than males (1.43 OR; 95% CI: 1.06 – 1.92).

**Table 1:** Characteristics of the study sample meeting past year (12-month) criteria for major depressive episode as a function of type of outpatient service use by gender (n = 1743)

	**Females**			**Males**		
	**N (%)**			**N (%)**	**OR**	**CI (95%)**
**Mental health service use**	600/1084 (55,4%)			346/659 (52,5%)	1.22	(0.92 – 1.36)
					**OR**	CI (95%)
Primary (FP only)	185 (17,1%)			84 (13,2%)	1.00	
FP and mental health specialist	275 (25,4%)			175 (26,6%)	1.41	(1.02-1.94)
Mental health specialist only	140 (12,9%)			87 (13,2%)	1.38	(0.95-1.99)
No contact	484 (44,6%)			313 (47,5%)	1.43	(1.06-1.92)
	**Females**			**Males**		
**Variables**	**N (%)**	**OR**	CI (95%)	**N (%)**	**OR**	CI (95%)
***Predisposing factors***						
**Age**						
15 – 24	109/249 (43,8)	1.64	(0.89 – 3.02)	51/137 (37,2)	0.49	(0.25 – 0.94)
25 – 44	288/471 (61,1)	3.30	(1.83 – 5.95)	156/279 (55,9)	1.04	(0.57 – 1.92)
45 – 64	185/308 (60,1)	3.15	(1.72 – 5.76)	113/195 (57,9)	1.13	(0.61 – 2.13)
65 +	18/56 (32,1)	Reference		27/49 (55,1)	Reference	
**Education**						
< secondary	122/273 (44,7)	Reference		83/164 (50,6)	Reference	
secondary	472/800 (59,0)	1.78	(1.35 – 2.35)	261/489 (53,4)	1.11	(0.78 – 1.57)
**Marital status**						
Widow/sep/div/single	348/616 (56,5)	1.12	(0.88 – 1.42)	205/375 (54,7)	1.23	(0.90 – 1.67)
Married/common-law	251/467 (53,7)	Reference		141/284 (49,6)	Reference	
***Enabling factors***						
**Household adjusted Income**						
Low	122/200 (61,0)	1.28	(0.94 – 1.76)	61/89 (68,5)	2.22	(1.37 – 3.58)
High	437/797 (54,8)	Reference		266/531 (50,1)	Reference	
**Social support**						
No (none/a little/some)	41/77 (53,2)	0.91	(0.57 – 1.45)	46/72 (63,9)	1.67	(1.01 – 2.76)
Yes (most/all the time)	559/1007 (55,5)	Reference		300/587 (51,1)	Reference	
**Accessibility**						
No	564/1033 (54,6)	Reference		327/628 (52,1)	Reference	
Yes	28/43 (65,1)	1.55	(0.82 – 2.94)	15/27 (55,6)	1.11	(0.52 – 2.39)
**Acceptability**						
No	480/882 (54,4)	Reference		275/514 (53,5)	Reference	
Yes	112/194 (57,7)	1.15	(0.84 – 1.57)	68/142 (47,9)	0.79	(0.55 – 1.15)
**Availability**						
No	542/1011 (53,6)	Reference		313/600 (52,2)	Reference	
Yes	50/64 (78,1)	3.02	(1.66 – 5.50)	30/56 (53,6)	1.05	(0.61 – 1.82)
***Need factors***						
**Chronic illness**						
No	45/120 (37,5)	Reference		33/115 (28,7)	Reference	
Yes	554/962 (57,6)	2.26	(1.53 – 3.34)	313/544 (57,5)	3.34	(2.15 – 5.17)
**Anxiety**						
No	377/746 (50,5)	Reference		190/418 (45,5)	Reference	
Yes	197/279 (70,6)	2.34	(1.74 – 3.14)	140/215 (65,1)	2.25	(1.60 – 3.16)
**Addictions**						
No	551/1009 (54.6)	Reference		288/549 (52,5)	Reference	
Yes	47/69 (68,1)	1.76	(1.05 – 2.95)	55/106 (51,9)	0.97	(0.64 – 1.47)
**Somatic symptoms**						
Less than 3	130/278 (46,8)	Reference		95/210 (45,2)	Reference	
3	470/806 (58,3)	1.59	(1.21 – 2.29)	251/449 (55,9)	1.54	(1.11 – 2.14)
**Past year suicide thought or attempt**						
No	409/819 (49,9)	Reference		222/435 (51,0)	Reference	
Yes	191/265 (72,1)	2.60	(1.93 – 3.52)	124/224 (55,4)	1.18	(0.86 – 1.64)
**Perceived mental health**						
Good/fair/poor	505/821 (61,5)	2.76	(2.07 – 3.69)	296/526 (56,3)	2.14	(1.45 – 3.17)
Very good/excellent	95/258 (36,8)	Reference		50/133 (37,6)	Reference	

The gender specific determinants associated with overall outpatient service use for mental health reasons are presented in Table 2. The results of the multivariate regression analyses showed that for both females and males with a MDE, the determinants associated with outpatient service use included higher education, marital status of being single, widowed, separated or divorced, the presence of a chronic illness and the presence of anxiety. Gender specific differences in determinants associated with outpatient service use included for males, lower adjusted household income (2.14 OR; 95% CI: 1.24–3.71), and for females, younger age [(25 to 44 years (2.77 OR; 95% CI: 1.34–5.74); 45 to 64 years (2.59 OR; 95% CI: 1.24–5.42)], the presence of social support (2.63 OR; 95% CI: (1.43 – 4.76), self-reported availability barriers (2.05 OR; 95% CI: 1.02–4.10), the presence of self-reported suicidal thoughts or attempt (2.80 OR; 95% CI: 1.92–4.09) and a poorer self-perceived mental health (1.80 OR; 95% CI: 1.27–2.55). Finally, the models explained 22% and 17% of the variability observed in outpatient service use for females and males.

**Table 2:** Gender specific multivariate analyses on the determinants of service use for mental health reasons among individuals with past year major depression (n = 1743)

	**Females**		**Males**	
**Variables**	Adjusted OR*	**CI (95%)**	Adjusted OR*	**CI (95%)**
***Predisposing factors***				
**Age**				
15 – 24	1.18	(0.54 – 2.54)	0.49	(0.21 – 1.13)
25 – 44	2.77	(1.34 – 5.74)	1.08	(0.51 – 2.30)
45 – 64	2.59	(1.24 – 5.42)	0.84	(0.39 – 1.80)
65 +	Reference		Reference	
**Education**	2.00	(1.39 – 2.88)	1.55	(1.00 – 2.40)
Secondary versus < secondary				
**Marital status**	1.43	(1.04 – 1.96)	1.67	(1.11 – 2.51)
Widow/sep/div/single versus Married/common-law				
***Enabling factors***				
**Household adjusted Income**	1.15	(0.78 – 1.70)	2.14	(1.24 – 3.71)
Low versus high				
**Social support**	2.63	(1.43 – 4.76)	0.83	(0.43 – 1.69)
Most/all the time versus None/a little/some				
**Accessibility **(Yes versus No)	0.87	(0.41 – 1.87)	1.41	(0.55 – 3.65)
**Acceptability **(Yes versus No)	0.93	(0.62 – 1.39)	0.67	(0.42 – 1.05)
**Availability **(Yes versus No)	2.05	(1.02 – 4.10)	0.69	(0.36 – 1.32)
***Need factors***				
**Chronic illness **(Yes versus No)	1.89	(1.18 – 3.00)	2.45	(1.48 – 4.08)
**Anxiety **(Yes versus No)	1.99	(1.40 – 2.83)	2.22	(1.48 – 3.34)
**Addictions **(Yes versus No)	1.39	(0.74 – 1.87)	0.98	(0.60 – 1.66)
**Somatic symptoms**	1.34	(0.96 – 1.87)	1.33	(0.91 – 1.96)
3 versus < 3				
**Past year suicide thought or attempt **(Yes versus No)	2.80	(1.92 – 4.09)	0.83	(0.55 – 1.24)
**Perceived mental health**	1.80	(1.27 – 2.55)	1.47	(0.92 – 2.37)
Good/fair/poor versus Very good/excellent				
**R square of Nagelkerke**	0.22		0.17	

*Adjusted for all other variables in model.

Among the respondents who met the criteria for major depression, 946 (54.3%) had consulted for mental health reasons in the year prior to interview. The gender specific determinants associated with type of outpatient service use among these users are presented in Table 3. The determinants associated with past year use of both GP/FP and mental health specialist services, as opposed to the use of a GP/FP only included, for males, poorer self perceived mental health status (2.45 OR; 95% CI: 1.00–6.01), the presence of an anxiety disorder (2.30 OR; 95% CI: 1.17–4.52) and self-reported acceptability barriers (0.32 OR; 95% CI: 0.14–0.74), whereas for females the determinants included marital status (1.83 OR; 95% CI: 1.17–2.85). When looking at the determinants associated with past year use of a mental health specialist only as opposed to GP/FP only, the determinants for males included the presence of self-reported accessibility barriers (0.07 OR; 95% CI: 0.01–0.74) and a MDE characterized by the presence of 3 somatic symptoms (0.35 OR; 95% CI: 0.16 – 0.77), whereas in females the determinants included self-reported acceptability (0.40 OR; 95% CI: 0.18–0.87) and availability barriers (3.47 OR; 95% CI: 1.30–9.26).

**Table 3:** Gender specific multinomial regression analyses on the determinants associated with type of outpatient service use among users (n = 946)

	**Females**				**Males**			
	**GP/FP and mental health specialist versus GP/FP only**		**Mental health specialist only versus GP/FP only**		**GP/FP and mental health specialist versus GP/FP only**		**Mental health specialist only versus GP/FP only**	
**Variables**	Adjusted OR*	**CI (95%)**	Adjusted OR*	**CI (95%)**	Adjusted OR*	**CI (95%)**	Adjusted OR*	**CI (95%)**
***Predisposing factors***								
**Age**								
15 – 24	2.17	(0.49 – 9.65)	1.04	(0.24 – 4.58)	0.98	(0.24 – 3.99)	2.68	(0.39 – 18.33)
25 – 44	2.15	(0.52 – 9.00)	1.12	(0.28 – 4.55)	0.63	(0.19 – 2.03)	2.39	(0.42 – 13.67)
45 – 64	1.88	(0.45 – 7.89)	0.42	(0.10 – 1.77)	1.14	(0.35 – 3.78)	2.77	(0.47 – 16.21)
65 +	Reference		Reference		Reference		Reference	
**Education**	1.56	(0.88 – 2.74)	0.99	(0.51 – 1.94)	1.99	(0.98 – 4.03)	1.50	(0.65 – 3.47)
Secondary versus < secondary								
**Marital status** Widow/sep/div/single versus Married/common-law	**1.83**	(1.17 – 2.85)	1.24	(0.72 – 2.15)	1.03	(0.51 – 2.05)	1.70	(0.75 – 3.85)
***Enabling factors***								
**Household adjusted Income**	0.96	(0.57 – 1.62)	1.12	(0.59 – 2.11)	0.80	(0.35 – 1.84)	0.62	(0.24 – 1.62)
Low versus high								
**Social support**	0.93	(0.38 – 2.29)	0.70	(0.22 – 2.25)	1.44	(0.50 – 4.14)	1.14	(0.32 – 4.09)
None/a little/some versus Most/all the time								
**Accessibility **(Yes versus No)	1.34	(0.47 – 3.79)	1.10	(0.29 – 4.20)	0.39	(0.09 – 1.77)	**0.07**	(0.01 – 0.74)
**Acceptability **(Yes versus No)	1.13	(0.65 – 1.96)	**0.40**	(0.18 – 0.87)	**0.32**	(0.14 – 0.74)	1.89	(0.79 – 4.54)
**Availability **(Yes versus No)	2.30	(0.94 – 5.61)	**3.47**	(1.30 – 9.26)	1.13	(0.37 – 3.41)	0.61	(0.15 – 2.48)
***Need factors***								
**Chronic illness **(Yes versus No)	1.11	(0.48 – 2.57)	0.58	(0.23 – 1.46)	1.37	(0.48 – 3.90)	0.71	(0.24 – 2.06)
**Anxiety **(Yes versus No)	1.22	(0.78 – 1.91)	1.17	(0.67 – 2.04)	**2.30**	(1.17 – 4.52)	2.16	(0.98 – 4.77)
**Addictions **(Yes versus No)	0.75	(0.34 – 1.63)	0.83	(0.31 – 2.20)	1.65	(0.69 – 3.97)	0.79	(0.29 – 2.20)
**Somatic symptoms**	0.94	(0.55 – 1.59)	0.60	(0.32 – 1.12)	1.00	(0.49 – 2.07)	**0.35**	(0.16 – 0.77)
3 versus < 3								
**Past year suicide thought or attempt **(Yes versus No)	1.23	(0.79 – 1.92)	0.88	(0.49 – 1.56)	1.39	(0.70 – 2.77)	1.32	(0.59 – 2.92)
**Perceived mental health**	0.59	(0.31 – 1.10)	0.69	(0.33 – 1.41)	**2.45**	(1.00 – 6.01)	0.48	(0.19 – 1.22)
Good/fair/poor versus Very good/excellent								
**R square of Nagelkerke**	0.26				0.11			

*Adjusted for all other variables in model. Significant effects are shown in bold.

## Discussion

This study contributes to the present literature by reporting on gender-specific determinants of outpatient service use for mental health reasons among respondents meeting criteria for a past year major depressive disorder in a large population based study. Moreover, this study compared variables associated with the exclusive and joint use of GP/FP and mental health specialist services for mental health reasons by users diagnosed with a MDE.

Our findings showed that close to 55% and 53% of females and males with a MDE in the past year consulted a professional for mental health reasons, which is similar to estimates in the National Comorbidity Survey Replication in the US, a large population based study, reporting service use rates reaching 56.8% among respondents with a major depressive disorder [21].

In this study, males and females with depression were just as likely to have consulted for a mental health reason. This finding concord with previous research suggesting that once men recognize their emotional problems, they are equally as likely as women to use mental health services [57]. Others have also reported that once a mental health problem reaches a certain threshold of severity, there is no significant difference between genders in the likelihood of seeking mental health care [20].

In females, mental health service use was greater in the age groups between 25 and 64 years as opposed to the 65 years and over age group, which has been similarly reported elsewhere [18,33]. Considering that most seniors consult a family physician annually, but that only a few seek treatment for their mental health problems and are treated, [15], better detection of mental health needs especially among older women is needed in primary care. The presence of social support was also associated with increased mental health service use in females, but not in males. In other general population based studies, the number of relatives was associated with decreased mental health service use in both males and females [46]. The difference in results may be due to the fact that we reported on a sample with depression and we included tangible (someone to take respondent to doctor, or carry out daily activities and help with meals), emotional and informational social support (have someone listen and advise them, confide in, someone that gives information and understands mental health problems). However, this suggest that the social network may play an important role on help-seeking behaviours of depressed individuals, and more so among women. Some have also suggested, people are less likely to support men in seeking mental health services as it goes against the prescribed role for men [58].

Further, the presence of self-reported suicidal thoughts or attempts and a poorer self- perceived mental health was also associated with mental health service use in females but not males. One study to date focusing on gender specific determinants of service use and suicidal ideation did not find any gender differences [59]. This study however reported on an older adult population and results may therefore not be comparable. It is however noteworthy that depressed males with suicidal ideation, controlling for other chronic and co-morbid conditions, were not more likely to seek mental health services. This has important implications for suicide prevention strategies.

In this study, mental health service use for males was positively associated with lower adjusted household income. This was similarly reported in a recent study on older adults with suicidal ideation in a public managed health care system [59]. In the study carried out by Ojeda and colleagues however (2006), household income was not associated with outpatient service use [18]. The differing results may in part be explained by the fact that we studied adjusted household income for number of people residing in the home as opposed to total household income, and in part by the fact that Canada has a public managed health care system which facilitates access to health services for vulnerable populations.

Previous large population studies have also reported on the determinants of general medical and mental health specialty services. Using data from the Epidemiologic Catchment Area (ECA), Burns and colleagues (2000) showed that the factors associated with specialty mental health service use as opposed to general medical services in individuals with depression were marital status of not being married, recurrent depression, poor perceived physical health and the presence of anxiety [14]. Others have found that men are more likely to use specialty mental health services for psychiatric disorders [22,35]. Moreover, it has been show that mental health specialist services versus general medical services treat individuals with more severe and complex depression [51,60]. Previous population based studies in Canada have also shown that receipt of specialty mental health care is associated with greater illness severity such as the presence of suicidal thoughts and persistent depression [23]. Further, individuals exhibiting more severe symptoms such as somatic symptoms, defined as ‘weight loss or weight gain’, ‘insomnia or hypersomnia’ and ‘fatigue or loss of energy’, the presence of comorbid depression and anxiety, and the presence of suicidal ideation were more likely to have consulted mental health specialist services as opposed to no use [41].

In this study, we examined the gender specific differences in the determinants associated with the use of specialty mental health service with and without the use of general medical services (i.e. GP/FP) as opposed to the use of GP/FP only. The results showed that for males, the determinants associated with the joint use of GP/FP and mental health specialist services as opposed to the use of a GP/FP only included poorer self perceived mental health status and the presence of an anxiety disorder. This was not observed in females suggesting that need factors are more important predictors of mental health specialist service use in males. This concords with previous findings suggesting that primary care physicians might show greater willingness to treat women while preferring to refer men to specialist services [22]. As our results also considered the presence of alcohol or drug abuse/dependence, future studies should focus on other factors that may explain this gender difference.

The results also showed that for males the presence of 3 or more somatic symptoms was associated with consulting GP/FP only as opposed to mental health specialist services only, but not associated with GP/FP and mental health specialist as opposed to GP/FP only. This is contrary to the report of Burns (2000) who did not find such an association [14]. Ten Have and colleagues however (2004), on a Dutch population based sample of adults with a lifetime major and minor depression, showed that individuals exhibiting more severe vegetative symptoms, defined as appetite, sleep and fatigue disturbance were more likely to have received specialised treatment [41]. The results however reported in these studies did not differentiate between males and females as was the case in our study.

The study findings also showed an association between self-reported barriers related to an unmet mental health care need and mental health care use. In males, the presence of accessibility barriers such as the inability to pay, scheduling conflicts and transportation was associated with consulting a GP/FP only as opposed to a mental health specialist. Further, the use of mental health professionals only in females as compared to the use of GP/FP only was positively associated with self-reported availability barriers such as waiting too long for services, help not available in area or at the time required. This suggests that females are more likely to consult other resources than a GP/FP in the presence of a mental health need.

Further, the presence of an unmet mental health need due to acceptability factors in females was associated with consulting a GP/FP only as opposed to a mental health professional only. This suggests that among females the decision to do without mental health care because of their attitudes towards the illness, health care providers or the health care system, negatively impacts the use of mental health specialists only which includes the services of psychiatrists, psychologists and social workers. This may be due to the lack of information regarding the type of mental health services offered by these professionals. In males, the presence of acceptability barriers was associated with consulting a GP/FP only as opposed to a mental health specialist and GP/FP, but not as opposed to a mental health specialist only. These results suggest that for males with acceptability issues consulting a GP/FP only when one is available is preferred to consulting mental health specialists. Others have similarly reported that the strongest predictor of help seeking from a general practitioner for males has been self-reported embarrassment [61].

The findings of this study need to be considered with the following limitations. The study sample excluded depressed people living in institutions or hospitalised, those living in the territories and homeless individuals. The results cannot be generalised to these populations as they may have more serious mental health issues and higher co-morbidities as well as different barriers in accessing health services. Mental health service use in this study did not include medications or hospitalisations, which might have underestimated past year mental health service use. Although depression and mental health service use were measured for their presence in the previous year, it is possible that the mental health service use reported was not for the depression itself but for another mental health problem. Finally, given the cross-sectional nature of the data, it is not possible discern the temporal sequence regarding past year mental health service use and reported barriers of unmet mental health needs. Longitudinal data with incident cases of depression with a study on pathways to care would be more informative. Further, although the results are based on data originating from 2002, there have not been any significant changes to the delivery of primary mental health care since then. Finally, the results of this study were based on secondary data analyses of the public data file of the CCHS1.2 survey and we could not control for a number of factors. One being, minority status and ethnic/racial groups which have been associated with mental health service use in some studies [18] but not others [36].

## Conclusion

In conclusion, the findings indicate that close to half of Canadian women and men with a probable depression did not use any outpatient mental health services in the past year. This represents a sizeable proportion of the population who has an unmet mental health care need. This is an important concern in the context of a public managed health care system as in Canada. It was noted that depressed males with suicidal ideation, controlling for other chronic and co-morbid conditions, were not more likely to seek mental health services. This has important implications for suicide prevention strategies among men. Further, depressed females aged 65 years and older were less likely to seek mental health services. This suggests that strategies designed to increase access to mental health services should focus on this vulnerable population. Finally, this study highlighted gender differences in the determinants of health service use and barriers to care that can serve as the basis for specific population strategies to increase utilization rates such as increased anti-stigma campaigns and the recognition of symptoms, and information regarding available effective treatments for depression.

## Competing interests

The authors declare that they have no competing interests.

## Authors’ contributions

SG, the corresponding author, contributed to the literature review and carried out the analyses and interpretation of data, and the writing of the paper. HMV participated in the study design, the interpretation of the results and the writing of the paper. MP participated in the interpretation and the writing of the paper. All authors (SG, HMV, MP) read and approved the final manuscript.

## Pre-publication history

The pre-publication history for this paper can be accessed here:

http://www.biomedcentral.com/1471-244X/14/135/prepub
